# Distribution and antimicrobial resistance profiles of vaginal bacteria in healthy dairy cows

**DOI:** 10.3389/fvets.2026.1761682

**Published:** 2026-02-13

**Authors:** Brandi M. Macleod, Theodoros Ntallaris, Jane M. Morrell, Ingrid Hansson, Csaba Varga

**Affiliations:** 1 College of Veterinary Medicine, University of Illinois at Urbana-Champaign, Urbana, IL, United States; 2 Department of Clinical Sciences, Swedish University of Agricultural Sciences, Uppsala, Sweden; 3 Department of Animal Biosciences, Swedish University of Agricultural Sciences, Uppsala, Sweden; 4 Department of Pathobiology, College of Veterinary Medicine, University of Illinois Urbana-Champaign, Urbana, IL, United States; 5 Carl R. Woese Institute for Genomic Biology, University of Illinois at Urbana-Champaign, Urbana, IL, United States

**Keywords:** antimicrobial resistance, clustering, dairy cows, microbiota, vaginal bacteria

## Abstract

**Introduction:**

Evaluating the vaginal bacterial isolates of dairy cows and their antimicrobial resistance patterns can improve animal health management practices and reduce the risk of transmitting antimicrobial-resistant pathogens within animal populations and from animals to humans.

**Materials and methods:**

This study characterized the culturable bacterial composition and antimicrobial resistance (AMR) profiles of vaginal bacteria isolated from healthy dairy cows at the Swedish Livestock Research Centre, Lövsta, Sweden. Unsupervised machine learning techniques, including Partitioning Around Medoids clustering and co-occurrence network analysis, investigated bacterial co-occurrences in the vaginal microbiota. Bacterial isolates were classified as resistant or wild type based on the epidemiological breakpoint values for inhibition zone diameters in the disk diffusion method defined by the European Committee on Antimicrobial Susceptibility Testing (EUCAST).

**Results:**

A total of 127 bacterial isolates representing 34 different species were isolated from the samples collected from the anterior vagina of 40 cows. *Histophilus somni*, *Streptococcus pluranimalium*, and *Escherichia coli* were the most prevalent species. The network analysis identified distinct microbial communities, revealing that *S. pluranimalium* and *H. somni* were central to microbial interactions within the vaginal microbiota. The disk diffusion susceptibility test identified *Staphylococcus* spp. isolates (*n* = 10) as resistant to penicillin (70%), tigecycline (50%), tetracycline (30%), and levofloxacin (30%), and were wild type for ciprofloxacin and trimethoprim-sulfamethoxazole. *Streptococcus* spp. isolates (*n* = 18) showed resistance to tetracycline (61.11%), tigecycline (27.78%), penicillin (22.22%), levofloxacin (11.11%), and trimethoprim-sulfamethoxazole (11.11%). *Escherichia coli* isolates (*n* = 11) exhibited resistance to ciprofloxacin (90.91%), tigecycline (81.82%), and levofloxacin (18.18%), and were wild type to trimethoprim-sulfamethoxazole.

**Conclusion:**

Our study highlights the importance of including commensal reproductive tract bacteria in AMR monitoring programs, as these species may serve as reservoirs of resistance genes and contribute to the spread of AMR within dairy herds.

## 1 Introduction

A balanced vaginal microbiota is important to maintain the reproductive health of dairy cows (1). Maintaining an appropriate composition is essential for preventing diseases such as endometritis and metritis, which are common in the periparturient period and can affect post-calving outcomes (2, 3). In healthy cows, a balanced commensal microbiota prevents the overgrowth of pathogenic microorganisms. However, disruptions in the vaginal microbiota, known as dysbiosis, can cause reproductive disorders and infections (4, 5). Herd management practices, such as maintaining hygiene during calving, breeding strategies, and genetic factors, can influence the microbial balance in the vaginal microbiota (6, 7). A well-balanced microbiota is linked to improved post-calving health, whereas dysbiosis is often associated with uterine diseases such as endometritis and metritis (8). Interestingly, cows with uterine diseases exhibit reduced bacterial diversity in their vaginal microbiota compared to healthy cows (9).

These commensal bacteria, including *Streptococcus* spp., *E. coli*, and *Staphylococcus* spp. are widespread in the genital tract of cattle. They are considered opportunistic pathogens that can cause infection when the cow’s natural defenses are compromised, such as during the postpartum period (10). *Streptococcus pluranimalium*, a common commensal bacterium, is considered an opportunistic pathogen in the anterior vagina of cows and is associated with various bovine reproductive diseases, including vaginitis, vulvitis, metritis, stillbirth, and abortion (11, 12). Additionally, *S. pluranimalium* has a zoonotic potential, causing endocarditis, brain abscesses, and septicemia in humans (13, 14). *Escherichia coli* is a natural part of a cow’s vaginal microbiota (15); however, certain strains can become pathogenic and cause reproductive problems such as reduced fertility or endometritis, especially postpartum (15). *Staphylococcus* species are a common and diverse component of the microbiota that colonizes the skin and mucous membranes (including the vagina) and the environment surrounding cows. In a healthy cow, these bacteria coexist with other microorganisms and generally do not cause disease (16). *Histophilus somni* is a common inhabitant of the normal bovine anterior vagina and other mucosal surfaces. It can act as an opportunistic pathogen, causing infections that may lead to infertility. The outcome depends on the virulence of the specific strain and the cow’s immune status (17).

The emergence of antimicrobial resistance (AMR) in livestock, including dairy cattle production, is a global health concern (18). It also has One Health implications, as human, animal, and environmental health are interconnected, and antimicrobial-resistant bacteria could be transmitted across these systems (19, 20). In addition, dairy cattle could serve as a reservoir for resistant bacteria, enabling transmission of these strains to humans through direct animal contact, environmental contamination, and the food chain via dairy products (21).

Antibiotic use in dairy farming exerts selection pressure on commensal and pathogenic bacteria of dairy cows, favoring the selection of antimicrobial-resistant strains (19). The emergence of antimicrobial-resistant bacteria on dairy farms contributes to increased treatment costs, higher morbidity, and an elevated incidence of treatment failure (22). Historically, antimicrobial resistance monitoring programs and research studies focused on assessing AMR in enteric pathogens and mastitis-causing bacteria in dairy production (23, 24), while the microbiota of the reproductive tract has received less attention. Further research studies are needed to understand the naturally occurring bacterial species in the reproductive tract of dairy cows and evaluate their AMR profiles, because these commensal bacteria may serve as hidden reservoirs for AMR determinants in dairy herds (4).

This study aimed to address this gap by characterizing the vaginal microbiota of healthy dairy cows, identifying bacterial species, and assessing their AMR profiles. Unsupervised machine learning techniques were used to construct bacterial co-occurrence networks to evaluate the relationships between bacterial species, and a clustering algorithm evaluated bacterial co-occurrences within the same animal. In addition, the predominant bacterial species identified within the networks were tested for resistance to commonly used antimicrobials. This study aimed to determine the distribution and antimicrobial resistance patterns of the bacteria in the vaginal microbiota of dairy cows and provide information that can guide health authorities in designing effective antimicrobial stewardship programs.

## 2 Materials and methods

### 2.1 Study population and sampling procedures

This study was conducted between March and May 2024 at a dairy research farm located at the Swedish Livestock Research Centre, Lövsta, Sweden. At this farm, around 300 cows are in production, and it is comparable with a Swedish commercial dairy farm regarding health status and husbandry. The cows were housed in an insulated loose-housing barn with rubber mats and sawdust-bedded cubicles. A total of 43 cows (55% Holstein, and 45% Swedish Red-and-White) were included in the study. Only healthy cows with no signs of vaginal discharge or clinical signs of disease at the time of sampling were included. The characteristics of cows included in the study are shown in Table 1.

**Table 1:** Characteristics of dairy cows included in a study of vaginal bacteria in healthy Swedish dairy cows.

Variable	Min	Max	Median	Mean
Age (months)	16.7	93	38.6	42
Lactation	0	6	2	2
7 days average milk production (liters)	97	223	122	141.8
Days after artificial insemination (AI)	0	10	4	3.6
Total AI to conception	1	4	1	1.6
Total inseminations	1	5	2	2.1
Milk production	16.8	63.6	35.8	37.2
Body composition score	2.7	4	3.6	3.5

Vaginal samples were collected using sterile, double-guarded uterine culture swabs. The vulva was first wiped with a disposable towel to minimize contamination. Swabs were inserted into the anterior vagina, carefully avoiding contact with the vulva and urethra, and rotated gently to collect mucus. Each swab was placed in an E-swab transport medium, labeled, and transported at room temperature to the laboratory for analysis, within a short time frame after collection to minimize any potential bias related to transport conditions.

Antimicrobial treatments within the previous year were reported for 14 cows. These antimicrobial treatments were as follows: 7 cows received dry cow therapy with a combination antimicrobial product containing active ingredients from beta-lactam and aminoglycoside antibiotics. Penicillin (beta-lactam) was used to treat mastitis (2 cows), foot rot (2 cows), reticuloperitonitis (1 cow), and infectious bursitis (1 cow).

### 2.2 Bacterial analyses

**Figure 1:**
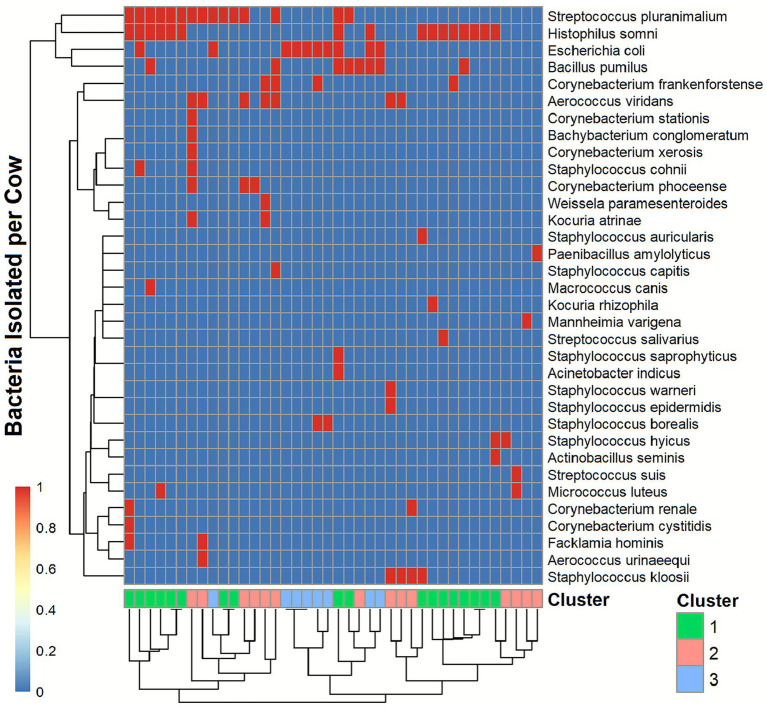
Cluster analysis of bacterial species in the vagina within cow groups based on Jaccard dissimilarity, considering the presence/absence of bacterial species. Rows represent bacterial species and columns represent individual cows. The heatmap indicates species presence (red) and absence (blue). Bacterial species and cows are grouped according to partitioning around medoids (PAM) clustering, with the three identified clusters indicated by color coding.

A variety of agar plates was selected to promote the growth and identification of as many different bacterial species as possible. Blood agar and Hematin agar (Swedish Veterinary Agency [SVA], Uppsala, Sweden), both non-selective media that support the growth of most culturable bacteria, were used for all samples. In addition, two selective agar media were used: Lactose purple agar (SVA) for identifying lactose-fermenting bacteria, and Mannitol salt agar (SVA) used for the analysis of *Staphylococcus* spp. Hematin agar plates were incubated at 37 °C with 5% CO2 for 24 + 24 h. Blood agar, lactose purple agar, and mannitol salt agar plates were incubated at 37 °C for 24 + 24 h. The plates were examined for bacterial growth after 24 h and 48 h by the same operator. The 24 + 24 h incubation protocol was used to capture both rapid and slow bacterial growth and to confirm colony morphology, hemolysis patterns, and carbohydrate fermentation characteristics that may not be evident after 24 h. The following criteria were used: no growth = no visible colonies after 48 h; poor growth = ≤10 colonies visible only on the first streak; sparse growth = > 10 colonies in the first streak and no growth in the second or third streak, intermediate growth = > 10 colonies in the first streak and growth in the second streak but not in the third streak; and heavy growth = > 10 colonies in the first streak and growth in both the second and third streaks (Figure 1). Visibly distinct colony types differing in shape, color, and size were re-cultured on blood agar plates at 37 °C with 5% CO2 for 24 h + 24 h to obtain a pure culture.

The isolates were identified at the species level by Matrix-Assisted Laser Desorption Ionisation Time of Flight Mass Spectrometry (MALDI-TOF MS). The mass spectra of the bacterial isolates were compared automatically with those of known bacterial strains in the database (Bruker Daltonics, Billerica, MA, USA). All isolates were preserved in cryotubes with brain-heart infusion broth containing 15% glycerol at −70 °C for subsequent antimicrobial susceptibility testing.

### 2.3 Antimicrobial susceptibility testing

Antimicrobial susceptibility testing was performed for *Escherichia coli*, *Streptococcus* spp., and *Staphylococcus* spp., as they were the dominant identified bacteria.

Disk-diffusion antimicrobial susceptibility testing was performed for all isolates according to the European Committee on Antimicrobial Susceptibility Testing (EUCAST) (25). Susceptibility to selected antibiotic substances was assessed with Thermo Scientific™ Oxoid™ 9 (Waltham, MA, USA) Antimicrobial Susceptibility discs. For *Streptococcus* spp. and *Staphylococcus* spp., the antibiotics: ciprofloxacin 5 μg (CIP), levofloxacin 5 μg (LEV), penicillin G 1 unit (PEN), tetracycline 30 μg (TET), tigecycline 15 μg (TGC), and trimethoprim-sulfamethoxazole 25 μg (SXT) (Thermo Fisher Scientific, Waltham, MA, USA) were tested. For *E. coli*, ciprofloxacin 5 μg (CIP), levofloxacin 5 μg (LEV), tetracycline 30 μg (TET), tigecycline 15 μg (TGC), and trimethoprim-sulfamethoxazole 25 μg (SXT). Disk diffusion inhibition zone measurements were interpreted using epidemiological cutoff values (ECOFFs) (26) defined by EUCAST. *Escherichia coli* ATCC 25922 and *Staphylococcus aureus* ATCC 29213 were used as quality control strains for gram-negative and gram-positive organisms, respectively.

Isolates with inhibition zone diameters below the ECOFFs were reported as ‘resistant’. The following inhibition zone diameter breakpoints (mm) for antimicrobials were used to define a resistant *E. coli* isolate: TGC (<18 mm), SXT (<11 mm), CIP (<22 mm), LEV (<19 mm).

For *Streptococcus* spp.: PEN (<18 mm), TGC (<19 mm), SXT (<15 mm), TET (<23 mm), LEV (<17 mm), and for *Staphylococcus* spp.: PEN (<26 mm), TGC (<19 mm), SXT (<14 mm), CIP (<17 mm), TET (<22 mm), LEV (<22 mm). This definition of “resistant” is used for surveillance (monitoring) purposes, and means that these isolates have acquired mechanisms that reduce susceptibility compared to wild-type bacteria, but such resistance does not always equate to clinical resistance (failure of therapy), which is defined using specific clinical breakpoints. Readers should therefore interpret ‘resistant’ in this context as a marker for AMR monitoring, not as a direct prediction of clinical outcome.

### 2.4 Statistical analysis

All analyses were conducted in R statistical software (Version 4.5.1 (2025-06-13)) within the RStudio platform (R Studio Version 1.4.1106© 2009–2021 RStudio, PBC), using the following packages: “binom,” dplyr,” “tidyr,” “ggplot2,” “igraph,” “tidygraph,” “ggraph,” “proxy,” “cluster,” and “pheatmap.”

Data of the bacterial isolates and susceptibility results were organized in Excel and imported into R for processing and analysis.

#### 2.4.1 Clustering of bacteria

To assess the clustering of bacteria within individual cows, the Partitioning Around Medoids (PAM) based on the Jaccard dissimilarity matrix was used (27). The silhouette width method was used to measure how well each observation fits within its assigned cluster compared to neighboring clusters.

The optimal number of clusters indicated a maximum average silhouette width at k = 10. However, because of the small sample size (40 cows), clustering at k = 10 resulted in several clusters containing only a single isolate, indicating over-partitioning and reduced cluster stability. To ensure robust and interpretable groupings, a reduced number of clusters was selected. A solution with k = 3 provided a balance between clustering performance and biological interpretability, yielding clusters with sufficient sample sizes for downstream analysis while preserving differences in bacterial community structure. Cluster-specific bacterial prevalence for the 3 clusters was then calculated and summarized.

#### 2.4.2 Bacteria co-occurrence network

To evaluate relationships between bacteria, a weighted undirected network was built using Jaccard similarity scores (28) calculated from a binary cow × bacteria matrix and was visualized. In the network the nodes represent the bacterial species, each characterized by a set of attributes, such as frequency: the number of cows in which the bacterium was detected; degree: the number of other bacterial species with which it co-occurs across all samples (indicating how many other bacteria it is connected to, but not necessarily within the same animal); and weighted degree: the strength of its connections, based on the Jaccard similarity, which reflects the similarity in bacterial co-occurrence patterns across all samples, not just within the same animal. The edges represent the connections between nodes (bacteria), indicating that the two bacterial species co-occur in the same sample across all cows. The strength of the edge, or its weight, is determined by the Jaccard similarity score, which measures how similar the occurrence patterns of the two bacterial species are across the samples. The weighted degree of each node was calculated based on the Jaccard similarity between bacterial pairs. The weighted degree reflects the strength of connections, with higher values indicating that a bacterium is more strongly associated with other bacteria in the network. The resulting similarity values were used to construct a weighted network, where the weights of the edges represent the degree of similarity between bacterial species. To assess the robustness of the network, and the stability of the bacterial co-occurrence network across different similarity cut-offs three different thresholds were applied to explore the impact of varying the Jaccard similarity cut-off on the network: Threshold 0: all bacterial pairs with any positive similarity (greater than 0) were included; Threshold 0.1: only bacterial pairs with a similarity greater than or equal to 0.1 were considered; and Threshold 0.2: bacterial pairs with a similarity greater than or equal to 0.2 were included. To assess whether the observed differences in weighted degree across the three thresholds were statistically significant, we performed both ANOVA and Kruskal-Wallis tests. The ANOVA was used to compare the means of weighted degree across thresholds, while the Kruskal-Wallis test was employed as a non-parametric alternative. The results were interpreted to determine whether the threshold choice significantly influenced the network structure.

## 3 Results

### 3.1 Bacterial analyses

A total of 181 isolates from 43 cows were tested with MALDI-TOF MS to identify bacteria at the species level. At least one bacterium was identified from 40 cows. A total of 127 bacterial isolates, representing 34 different species, were distinguished from vaginal samples of 40 cows (Table 2). The most common species identified were: *Histophilus somni* (28 isolates), *Streptococcus pluranimalium* (20), *Escherichia coli* (11), *Bacillus pumilus* (10), and *Aerococcus viridans* (8). No identification was possible, or no peaks were identified in 54 isolates.

**Table 2:** Distribution of bacteria isolated from the anterior vagina of 40 healthy Swedish dairy cows.

Bacteria list	Number (n)	Percent (n/N^a^)
*Histophilus somni*	28	15.47
*Streptococcus pluranimalium*	20	11.05
*Escherichia coli*	11	6.08
*Bacillus pumilus*	10	5.52
*Aerococcus viridans*	8	4.42
*Facklamia hominis*	5	2.76
*Staphylococcus kloosii*	5	2.76
*Corynebacterium frankenforstense*	4	2.21
*Corynebacterium phoceense*	4	2.21
*Actinobacillus seminis*	2	1.1
*Corynebacterium renale*	2	1.1
*Kocuria atrinae*	2	1.1
*Micrococcus luteus*	2	1.1
*Staphylococcus borealis*	2	1.1
*Staphylococcus cohnii*	2	1.1
*Staphylococcus hyicus*	2	1.1
*Acinetobacter indicus*	1	0.55
*Aerococcus urinaeequi*	1	0.55
*Bachybacterium conglomeratum*	1	0.55
*Corynebacterium cystitidis*	1	0.55
*Corynebacterium stationis*	1	0.55
*Corynebacterium xerosis*	1	0.55
*Kocuria rhizophila*	1	0.55
*Macrococcus canis*	1	0.55
*Mannheimia varigena*	1	0.55
*Paenibacillus amylolyticus*	1	0.55
*Staphylococcus auricularis*	1	0.55
*Staphylococcus capitis*	1	0.55
*Staphylococcus epidermidis*	1	0.55
*Staphylococcus saprophyticus*	1	0.55
*Streptococcus salivarius*	1	0.55
*Streptococcus suis*	1	0.55
*Streptococcus warneri*	1	0.55
*Weissella paramesenteroides*	1	0.55
No identification possible	35	19.34
No peaks	19	10.5

a *N* = 181.

### 3.2 Cluster analysis

The Partitioning Around Medoids (PAM) method grouped cows based on Jaccard dissimilarity, considering the presence/absence of bacterial species, into 3 groups of cows (clusters) with similar microbial compositions (Figure 1 and Supplementary Table 1). Cluster 1 included 18 cows. The most common bacteria isolated from cows were *Histophilus somni* (83%), *Streptococcus pluranimalium* (56%), and *Escherichia coli* (11%). Cluster 2 included 14 cows; the most common bacteria isolated were *Aerococcus viridans* (50%), *Streptococcus pluranimalium* (29%), *Staphylococcus kloosii* (21%), and *Corynebacterium phoceense* (21%). Cluster 3 included 8 cows; the most common isolated bacteria were *Escherichia coli* (100%), *Bacillus pumilus* (25%), and *Staphylococcus borealis* (25%).

### 3.3 Network analysis

**Figure 2:**
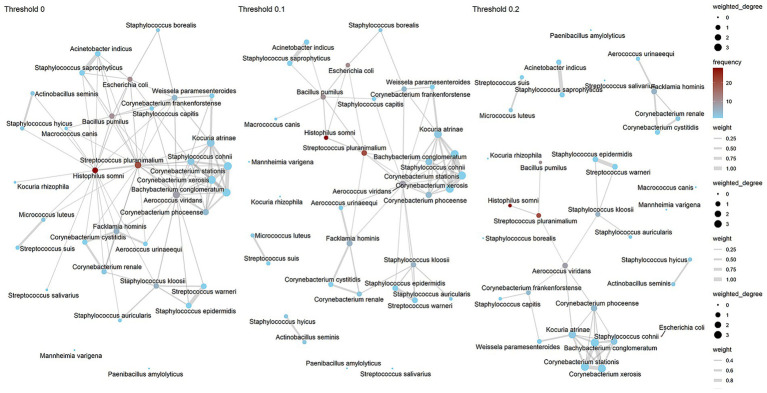
Jaccard co-occurrence network analysis of vaginal bacterial isolates from 40 healthy Swedish dairy cows at three thresholds (0, 0.1, and 0.2). Nodes represent bacterial taxa and are colored by frequency of occurrence (red = higher; blue = lower), while node size reflects weighted degree. Edges indicate co-occurrence relationships, with edge thickness corresponding to association strength.

The results of the bacterial co-occurrence networks with the three different thresholds (0, 0.1, and 0.2) are presented in Figure 2 and Supplementary Table 2. In all three networks, three main nodes were identified: *Streptococcus pluranimalium*, *Histophilus somni*, and *Aerococcus viridans*, which were positioned centrally in the network; they also had a high frequency and a high degree of connectivity. Strong edges, indicating higher co-occurrences, were identified in all three networks among: *Streptococcus pluranimalium* and *Histophilus somni; Staphylococcus epidermidis and Staphylococcus warneri; and Corynebacterium stationis and Bachybacterium conglomeratum.*

Both the Kruskal-Wallis (chi-squared = 4.1659, df = 2, *p*-value = 0.1246) and ANOVA (*F* value = 1.048, p-value is 0.355) tests were not significant, and visually the weighted degree of isolates were not very different (Supplementary Figure 1). These findings indicate that the changes in threshold did not lead to statistically significant differences in the weighted degree of bacterial species in the co-occurrence network. In addition, the network structure was robust to variations in the threshold, and the choice of threshold did not significantly change the relationships between bacterial species in the vaginal bacterial isolates.

### 3.4 Antimicrobial susceptibility

A total of 10 *Staphylococcus* spp. (3 isolates of *S. kloosii*, 2 isolates each of *S. hyicus and S.cohnii,* and 1 isolate each of *S. borealis, S. capitis*, and *S. epidermidis*), 18 *Streptocccus* spp. (15 isolates of *S. pluranimalium*, and 1 isolate each of *S. luteinesis*, *S. salivarius,* and *S. warneri*), and 11 *E. coli* isolates were tested for susceptibility to antimicrobials (Table 3).

**Table 3:** Distribution of antimicrobial resistance among bacteria isolated from the anterior vagina of 40 healthy dairy cows.

Bacteria	Antimicrobial^a^	Resistant	Sensitive	% Resistant	95% CI
*Streptococcus* spp. (*n* = 18)	PEN	4	14	22.22	6.41–47.64
	TGC	5	13	27.78	9.69–53.48
	SXT	2	16	11.11	1.38–34.71
	TET	11	7	61.11	35.75–82.70
	LEV	2	16	11.11	1.38–34.71
*Staphylococcus* spp.(*n* = 10)	PEN	7	3	70.00	34.75–93.33
	TGC	5	5	50.00	18.71–81.29
	SXT	0	10	0.00	0.00–30.85
	CIP	0	10	0.00	0.00–30.85
	TET	3	7	30.00	6.67–65.25
	LEV	3	7	30.00	6.67–65.25
*Escherichia coli* (*n* = 11)	TGC	9	2	81.82	48.22–97.72
	SXT	0	11	0.00	0.00–28.49
	CIP	10	1	90.91	58.72–99.77
	LEV	9	2	18.18	2.28–51.78

a CIP, Ciprofloxacin, Levofloxacin (LEV), Penicillin (PEN), Tetracycline (TET), Tigecycline (TGC), Trimethoprim-Sulfamethoxazole (SXT).

Among *Staphylococcus* spp. (*n* = 10), 70% were resistant to penicillin, 50% to tigecycline, and 30% to tetracycline and levofloxacin. Whereas all tested isolates of *Staphylococcus* spp. were susceptible to ciprofloxacin and trimethoprim-sulfamethoxazole.

Among the *Streptococcus* spp. (*n* = 18) isolates, 61.11% were identified as resistant to tetracycline, 27.78% to tigecycline, 22.22% to penicillin, and 11.11% to levofloxacin, and trimethoprim-sulfamethoxazole.

Among *E. coli* isolates (*n* = 11), a high level of resistance was identified to ciprofloxacin (90.91%) and tigecycline (81.82%), and 18.18% showed resistance to levofloxacin, whereas all isolates were susceptible to trimethoprim-sulfamethoxazole.

**Figure 3:**
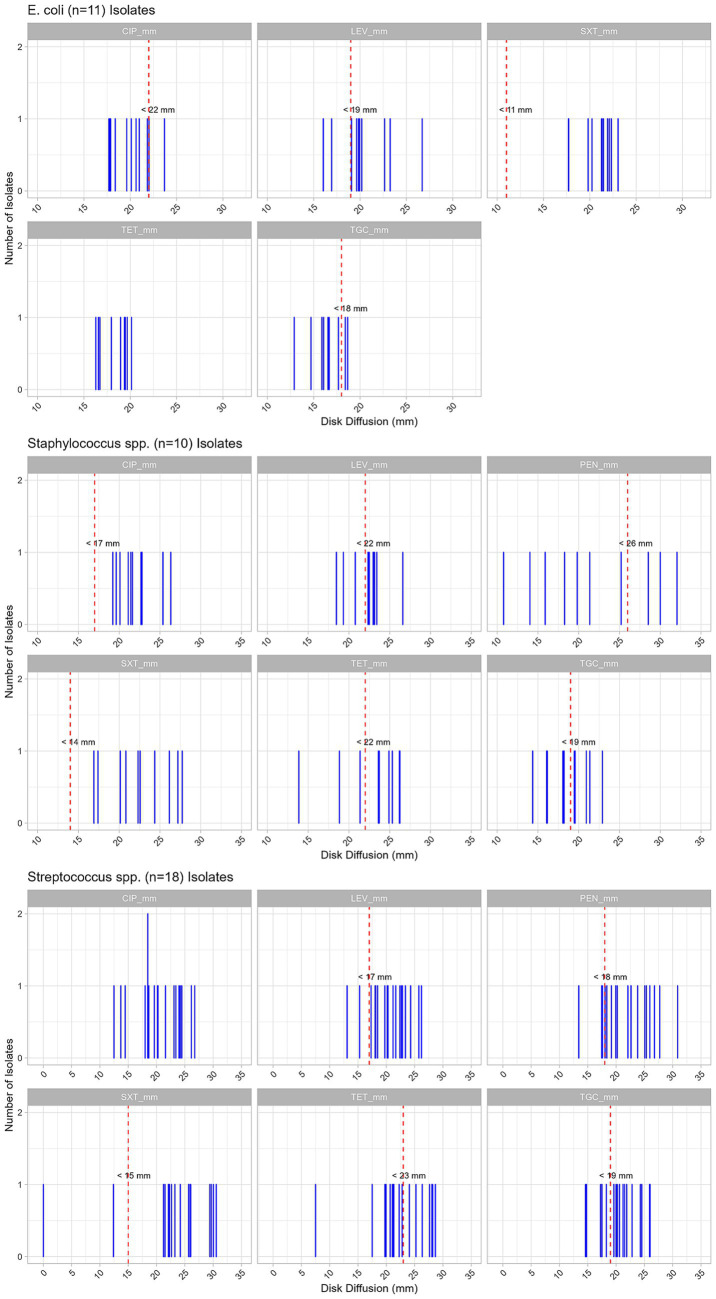
Distribution of inhibition zone diameters for antimicrobial susceptibility testing of *Streptococcus* spp., *Staphylococcus* spp., and *Escherichia coli* isolates from vaginal samples of dairy cattle. Individual blue lines represent inhibition zone diameters for each isolate. Red dashed lines indicate the epidemiological cut-off (breakpoint) values used to distinguish wild-type from non–wild-type (resistant) isolates. Antimicrobials tested include ciprofloxacin (CIP), levofloxacin (LEV), penicillin (PEN), tetracycline (TET), tigecycline (TGC), and trimethoprim–sulfamethoxazole (SXT).

In addition to presenting the proportion of resistance to antimicrobials as a binomial outcome (resistant/susceptible) for the tested antimicrobials across *Streptococcus* spp., *Staphylococcus* spp., and *E. coli* isolates, we also described their inhibition zone diameter characteristics (mean, median, minimum, and maximum) (Table 4), and illustrated the distribution of inhibition zone values for each isolate in Figure 3. The wild-type isolates were susceptible to the drug (above the resistance breakpoint), while the resistant isolates showed reduced susceptibility (below the resistance breakpoint). To provide a general overview of the resistance patterns of bacterial populations, the population of bacteria was characterized as resistant if the mean inhibition zone diameter was below the resistance breakpoint, and wild type if it was above (Table 4).

**Table 4:** Inhibition zone measurements and resistance classifications for antimicrobials in *Streptococcus* spp., *Staphylococcus* spp., and *E. coli* isolates from the anterior vagina of 40 healthy dairy cattle.

Bacteria/Antimicrobials	Breakpoints (mm)	Mean (mm)	Median (mm)	Minimum (mm)	Maximum (mm)	Interpretation (mean)
*Escherichia coli* (*n* = 11)						
Ciprofloxacin (CIP)	<22 mm	20.06	20.10	17.70	23.69	Resistant (below breakpoint)
Levofloxacin (LEV)	<19 mm	20.30	19.82	16.03	26.70	Wild type (above breakpoint)
Tetracycline (TET)	NA	18.17	18.45	16.28	20.14	NA^a^
Tigecycline (TGC)	<18 mm	16.41	16.55	12.90	18.67	Resistant (below breakpoint)
Trimethoprim-Sulfamethoxazole (SXT)	<11 mm	20.81	21.32	17.70	23.05	Wild type (above breakpoint)
*Staphylococcus* spp. (*n* = 10)						
Ciprofloxacin (CIP)	<17 mm	22.03	21.54	19.22	26.33	Wild type (above breakpoint)
Levofloxacin (LEV)	<22 mm	22.26	22.73	18.46	26.60	Wild type (above breakpoint)
Penicillin (PEN)	<26 mm	21.59	20.59	10.79	32.05	Resistant (below breakpoint)
Tetracycline (TET)	<22 mm	23.02	24.31	13.85	26.25	Wild type (above breakpoint)
Tigecycline (TGC)	<19 mm	18.70	18.83	14.35	22.90	Resistant (below breakpoint)
Trimethoprim-Sulfamethoxazole (SXT)	<14 mm	22.56	22.44	16.90	27.73	Wild type (above breakpoint)
*Streptococcus* spp. (*n* = 18)						
Ciprofloxacin (CIP)	NA	20.43	20.21	12.48	26.75	NA
Levofloxacin (LEV)	<17 mm	20.60	20.75	13.07	26.20	Wild type (above breakpoint)
Penicillin (PEN)	<18 mm	22.06	22.36	13.41	30.87	Wild type (above breakpoint)
Tetracycline (TET)	<23 mm	22.42	21.80	7.50	28.67	Resistant (below breakpoint)
Tigecycline (TGC)	<19 mm	20.59	20.41	14.59	26.00	Wild type (above breakpoint)
Trimethoprim-Sulfamethoxazole (SXT)	<15 mm	23.04	23.72	0.00	30.55	Wild type (above breakpoint)

a NA: Not available due to the absence of established epidemiological cut-off values for resistance interpretation.

## 4 Discussion

This study determined the vaginal microbiota of healthy dairy cows and highlighted the diversity of bacterial species contained therein and their antimicrobial resistance (AMR) profiles. A total of 127 isolates representing 34 different bacterial species were identified, with *Histophilus somni*, *Streptococcus pluranimalium*, and *Escherichia coli* being the most prevalent species. The bacterial species isolated from the same cow were evaluated using an unsupervised machine learning technique, Partitioning Around Medoids (PAM) (27), which identified groups of cows with similar microbial compositions. The co-occurrence network analysis identified *Streptococcus pluranimalium*, *Histophilus somni*, and *Aerococcus viridans* as central nodes of the network, having a high frequency and a degree of connectivity. Antimicrobial resistance determinants of the main bacterial species were assessed to identify resistant and wild-type isolates, providing information that can aid health authorities in the implementation of antimicrobial stewardship programs.

As expected, most bacteria isolated from healthy dairy cows in this study were commensal, representing the normal microbiota of the reproductive tract. However, previous studies have shown that under certain conditions, such as environmental stressors or reduced immunity, particularly postpartum, these commensal bacteria can become pathogenic and contribute to infections of the reproductive tract (29).

*Streptococcus pluranimalium* was identified as an important bacterium in this study. The high frequency, centrality, and connectivity of *Streptococcus pluranimalium* in the co-occurrence network suggest that *S. pluranimalium* might have an influential role in the vaginal microbiota of healthy dairy cows, and may influence its composition and interactions with other bacterial species. *Streptococcus pluranimalium* isolates were clustered with *Histophilus somni* and *Aerococcus viridans* within the same cow, indicating that these bacterial species may share similar ecological niches or environmental conditions that promote their co-existence. Among the *S. pluranimalium* isolates, resistance to tetracycline, tigecycline, penicillin, and levofloxacin was observed. However, resistance was defined by epidemiological breakpoints and might not always equate to clinical resistance (failure of therapy). Nevertheless, these resistance patterns might limit the treatment effectiveness of infections caused by *S. pluranimalium.* Especially, resistance to penicillin is concerning as it is the first-choice antimicrobial to treat *Streptococcus* spp. infections. Resistance to tetracycline is also worrying, as it is a commonly used antibiotic in veterinary medicine to treat reproductive infections in some countries (30, 31). Additionally, the presence of isolates resistant to multiple antimicrobials suggests that *S. pluranimalium* could act as a reservoir of multidrug-resistance determinants and could facilitate the spread of resistant genes within the herd and across environments. The zoonotic potential of *S. pluranimalium* should also be considered, as previous studies described infections in humans with this bacterium (32).

In this study, *E. coli* was isolated from the anterior vagina of dairy cows. *E. coli* is considered a commensal organism in the gastrointestinal tract of cattle, where intestinal *E. coli* does not normally cause infections. However, under certain conditions, such as environmental stressors, impaired immunity, or postpartum uterine infections, certain *E. coli* strains can become pathogenic, causing extra-intestinal infections such as metritis and endometritis (33). In addition, a previous study reported that the reproductive tract could be populated via ascending colonization from intestinal *E. coli* (34). Interestingly, our study identified a cluster of 8 cows from which *E. coli* was isolated, along with *Bacillus pumilus* (present in 25% of cows) and *Staphylococcus borealis* (also present in 25% of cows). This co-occurrence may reflect the influence of similar environmental conditions, health status, or time of the estrus cycle, or other factors that promoted their co-occurrence (35). In addition, our study has found a high resistance to ciprofloxacin (91%) and tigecycline (80%) in *E. coli* isolates, which is concerning, considering the lower resistance described in previous antimicrobial resistance surveillance reports in Sweden (36). However, comparing our study to those reports should be made with caution, as differences in sampling design, sampling source, and antimicrobial susceptibility methods exist. The emergence of resistance to fluoroquinolones (e.g., CIP) and tetracyclines (e.g., TGC) could limit treatment options and efficacy for *E. coli* infections in dairy cattle. The detection of fluoroquinolone resistance in *Escherichia coli* isolated from the vaginal microbiota of cows is concerning, as plasmid-mediated quinolone resistance (PMQR) genes represent a public health concern and are rarely reported in cattle. Future genomic studies are warranted to investigate this finding to elucidate the underlying resistance mechanisms.

In this study, several *Staphylococcus* spp., including *S. kloosii*, *S. hyicus*, *S. cohnii*, *S. borealis*, *S. capitis*, *S. warneri,* and *S. epidermidis*, were isolated from the anterior vagina of dairy cows. Previous studies described that *Staphylococcus* spp. are prevalent commensal non-pathogenic bacterial genera found in the vaginal microbiota of healthy dairy cows, along with *Bacillus* and *Streptococcus* species (4). However, in certain settings, especially following parturition (calving) or when the host’s immune defenses are compromised, opportunistic *Staphylococcus* spp. could become pathogenic, and they could be isolated from cows with purulent vaginal discharge (a sign of endometritis) and other uterine infections, often in polymicrobial infections alongside other common pathogens like *E. coli* and *Trueperella pyogenes* (37). Our study is in agreement with this observation, since *Staphylococcus borealis* was isolated together with *Escherichia coli* and *Bacillus pumilus* in Cluster 3, and was present in 25% of cows in the group. In addition, a high level of resistance was identified for penicillin and tigecycline, which are commonly used in veterinary medicine to treat infections of the reproductive tract of cattle (38). This finding agrees with several previous investigations that identified a high resistance to penicillin in *Staphylococcus* spp. isolated from dairy cows (39–41).

The high resistance of isolates to commonly used antimicrobials in veterinary medicine, even in the absence of clinical disease, and the presence of multidrug-resistant (resistant to 3 or more antimicrobial classes) (42) isolates in the vaginal microbiota of healthy dairy cows are concerning and recommend updating current antimicrobial stewardship programs.

The high resistance to antimicrobials in both Gram-positive (*Streptococcus* spp. and *Staphylococcus* spp.) and Gram-negative (*E. coli*) bacteria suggests that the reproductive tract may act as a reservoir for resistant bacteria. Bacteria with resistance genes and mutations could spread to the farm environment and could pose a zoonotic transmission risk via direct animal-to-human contact or indirectly via dairy products or fomites (43).

Our study finding suggests that monitoring programs should include bacterial species of the vaginal microbiota, especially *Streptococcus pluranimalium* and *E. coli,* as current monitoring efforts focus on enteric and mastitis pathogens. At the same time, reproductive tract commensals are neglected. Integrating surveillance of the vaginal microbiota into existing AMR monitoring programs is suggested to identify reservoirs of AMR and identify emerging antimicrobial-resistant strains. As these bacteria could be transmitted to humans, a One Health framework should be followed when designing stewardship programs to mitigate the impact of AMR across species and environments.

Before interpreting our study results, some limitations should be considered. Our study used epidemiological breakpoints to characterize resistance to various antimicrobials in bacteria of the vaginal microbiota of healthy dairy cows; therefore, the findings reflect resistance phenotypes rather than predicted clinical treatment outcomes. Moreover, most antimicrobials included in the susceptibility testing panel are not authorized for use in food-producing animals in Sweden, and the observed susceptibility patterns should be interpreted as sentinel indicators within a One Health surveillance framework. In addition, our study was a pilot study, with a small sample size and a single farm. The diversity in farm conditions, management practices, and local environmental factors could all impact the composition of the vaginal microbiota and antimicrobial resistance. Future studies that include several dairy farms across broader geographic regions are needed to understand the persistence and occurrence of resistant strains and changes over time on these farms. Also, further studies are needed to understand the role of factors impacting the selection of antimicrobial-resistant strains, including antimicrobial use. Moreover, studies should evaluate alternatives to antimicrobials, including probiotics, vaccines, or bacteriophage therapy, to reduce reliance on antibiotics. Lastly, metagenome and resistome analyses should follow up our study to further evaluate the vaginal microbiota.

## 5 Conclusion

This study described the complex interactions between bacterial species in the vaginal microbiota, with *Streptococcus pluranimalium* and *Histophilus somni* identified as central nodes in the co-occurrence network. The identification of AMR patterns among *E. coli*, *Staphylococcus* spp., and *Streptococcus* spp. isolates emphasizes the need for integrative AMR monitoring programs that consider the entire microbiota of the anterior vagina of dairy cows. The identified resistance to commonly used antimicrobials in veterinary medicine and the presence of multidrug-resistant isolates are concerning and suggest that alternatives to antimicrobials are needed, including improved biosecurity and farm management practices that reduce the bacterial load on dairy farms and the need for antimicrobial treatments.

This study emphasizes the integration of reproductive tract microbiota into AMR surveillance programs for dairy cattle. Understanding the relationships between commensal and opportunistic pathogenic bacteria, their antimicrobial resistance profiles, and the factors influencing the selection of resistance traits will aid animal health stakeholders in developing effective antimicrobial stewardship strategies in the dairy industry.

## Data Availability

The original contributions presented in the study are included in the article/Supplementary material, further inquiries can be directed to the corresponding author.
