# Supplementary material for: Distribution and antimicrobial resistance profiles of vaginal bacteria in healthy dairy cows
Source: Front Vet Sci. 2026 Feb 13;13:1761682. doi: 10.3389/fvets.2026.1761682 (PMC12945782; doi:10.3389/fvets.2026.1761682)

Supplementary Material

**Supplementary Table 1.** Number of identified bacteria within groups of dairy cows (clusters).

| Clusters |  | Cluster 1 | Cluster 2 | Cluster 3 |
| --- | --- | --- | --- | --- |
| Number of cows |  | **18** | **14** | **8** |
| Bacteria & Number | *Acinetobacter indicus* | 1 | 0 | 0 |
|  | *Actinobacillus seminist* | 1 | 0 | 0 |
|  | *Aerococcus urinaeequi* | 0 | 1 | 0 |
|  | *Aerococcus viridans* | 0 | 7 | 0 |
|  | *Bachybacterium conglomeratum* | 0 | 1 | 0 |
|  | *Bacillus pumilus* | 4 | 2 | 2 |
|  | *Corynebacterium cystitidis* | 1 | 0 | 0 |
|  | *Corynebacterium frankenforstense* | 1 | 2 | 1 |
|  | *Corynebacterium phoceense* | 0 | 3 | 0 |
|  | *Corynebacterium renale* | 1 | 1 | 0 |
|  | *Corynebacterium stationis* | 0 | 1 | 0 |
|  | *Corynebacterium xerosis* | 0 | 1 | 0 |
|  | *Escherichia coli* | 2 | 0 | 8 |
|  | *Facklamia hominis* | 1 | 1 | 0 |
|  | *Histophilus somni* | 15 | 0 | 1 |
|  | *Kocuria atrinae* | 0 | 2 | 0 |
|  | *Kocuria rhizophila* | 1 | 0 | 0 |
|  | *Macrococcus canis* | 1 | 0 | 0 |
|  | *Mannheimia varigena* | 0 | 1 | 0 |
|  | *Micrococcus luteus* | 1 | 1 | 0 |
|  | *Paenibacillus amylolyticus* | 0 | 1 | 0 |
|  | *Staphylococcus auricularis* | 1 | 0 | 0 |
|  | *Staphylococcus borealis* | 0 | 0 | 2 |
|  | *Staphylococcus capitis* | 0 | 1 | 0 |
|  | *Staphylococcus cohnii* | 1 | 1 | 0 |
|  | *Staphylococcus epidermidis* | 0 | 1 | 0 |
|  | *Staphylococcus hyicus* | 1 | 1 | 0 |
|  | *Staphylococcus kloosii* | 1 | 3 | 0 |
|  | *Staphylococcus saprophyticus* | 1 | 0 | 0 |
|  | *Staphylococcus warneri* | 0 | 1 | 0 |
|  | *Streptococcus pluranimalium* | 10 | 4 | 1 |
|  | *Streptococcus salivarius* | 1 | 0 | 0 |
|  | *Streptococcus suis* | 0 | 1 | 0 |
|  | *Weissela paramesenteroides* | 0 | 1 | 0 |

**Supplementary Table 2.** Network node metrics of bacteria isolated from the anterior vagina of dairy cows.

| Bacteria | Frequency | Threshold 0 | | Threshold 0.1 | | Threshold 0.2 | |
| --- | --- | --- | --- | --- | --- | --- | --- |
|  |  | **Degree** | **Weighted degree** | **Degree** | **Weighted degree** | **Degree** | **Weighted degree** |
| *Acinetobacter indicus* | 1 | 5 | 1.354 | 2 | 1.125 | 1 | 1.000 |
| *Actinobacillus seminis* | 2 | 2 | 0.563 | 1 | 0.500 | 1 | 0.500 |
| *Aerococcus urinaeequi* | 1 | 3 | 0.710 | 2 | 0.643 | 1 | 0.500 |
| *Aerococcus viridans* | 8 | 16 | 2.667 | 15 | 2.595 | 5 | 1.202 |
| *Bachybacterium conglomeratum* | 1 | 7 | 3.543 | 6 | 3.476 | 5 | 3.333 |
| *Bacillus pumilus* | 10 | 9 | 1.273 | 7 | 1.111 | 1 | 0.211 |
| *Corynebacterium cystitidis* | 1 | 4 | 1.129 | 2 | 1.000 | 2 | 1.000 |
| *Corynebacterium frankenforstense* | 4 | 9 | 1.398 | 5 | 1.122 | 3 | 0.722 |
| *Corynebacterium phoceense* | 4 | 7 | 1.875 | 7 | 1.875 | 6 | 1.750 |
| *Corynebacterium renale* | 2 | 5 | 1.155 | 3 | 1.033 | 2 | 0.833 |
| *Corynebacterium stationis* | 1 | 7 | 3.543 | 6 | 3.476 | 5 | 3.333 |
| *Corynebacterium xerosis* | 1 | 7 | 3.543 | 6 | 3.476 | 5 | 3.333 |
| *Escherichia coli* | 11 | 8 | 1.035 | 4 | 0.667 | 0 | 0.000 |
| *Facklamia hominis* | 5 | 6 | 1.650 | 5 | 1.592 | 3 | 1.333 |
| *Histophilus somni* | 28 | 18 | 1.521 | 3 | 0.622 | 1 | 0.292 |
| *Kocuria atrinae* | 2 | 9 | 3.132 | 8 | 3.069 | 7 | 2.869 |
| *Kocuria rhizophila* | 1 | 1 | 0.063 | 0 | 0.000 | 0 | 0.000 |
| *Macrococcus canis* | 1 | 3 | 0.254 | 1 | 0.125 | 0 | 0.000 |
| *Mannheimia varigena* | 1 | 0 | 0.000 | 0 | 0.000 | 0 | 0.000 |
| *Micrococcus luteus* | 2 | 3 | 0.621 | 1 | 0.500 | 1 | 0.500 |
| *Paenibacillus amylolyticus* | 1 | 0 | 0.000 | 0 | 0.000 | 0 | 0.000 |
| *Staphylococcus auricularis* | 1 | 2 | 0.313 | 1 | 0.250 | 1 | 0.250 |
| *Staphylococcus borealis* | 2 | 2 | 0.400 | 2 | 0.400 | 0 | 0.000 |
| *Staphylococcus capitis* | 1 | 4 | 0.585 | 3 | 0.518 | 1 | 0.250 |
| *Staphylococcus cohnii* | 2 | 9 | 2.491 | 7 | 2.342 | 5 | 2.083 |
| *Staphylococcus epidermidis* | 1 | 3 | 1.393 | 3 | 1.393 | 2 | 1.250 |
| *Staphylococcus hyicus* | 2 | 2 | 0.559 | 1 | 0.500 | 1 | 0.500 |
| *Staphylococcus kloosii* | 5 | 6 | 1.225 | 5 | 1.172 | 4 | 0.972 |
| *Staphylococcus saprophyticus* | 1 | 5 | 1.354 | 2 | 1.125 | 1 | 1.000 |
| *Streptococcus pluranimalium* | 20 | 20 | 2.096 | 7 | 1.252 | 3 | 0.724 |
| *Streptococcus salivarius* | 1 | 1 | 0.063 | 0 | 0.000 | 0 | 0.000 |
| *Streptococcus suis* | 1 | 1 | 0.500 | 1 | 0.500 | 1 | 0.500 |
| *Streptococcus warneri* | 1 | 3 | 1.393 | 3 | 1.393 | 2 | 1.250 |
| *Weissela paramesenteroides* | 1 | 3 | 0.893 | 3 | 0.893 | 2 | 0.750 |

**Supplementary Figure 1.** Comparison of weighted degree distributions in vaginal bacterial co-occurrence networks across Jaccard thresholds


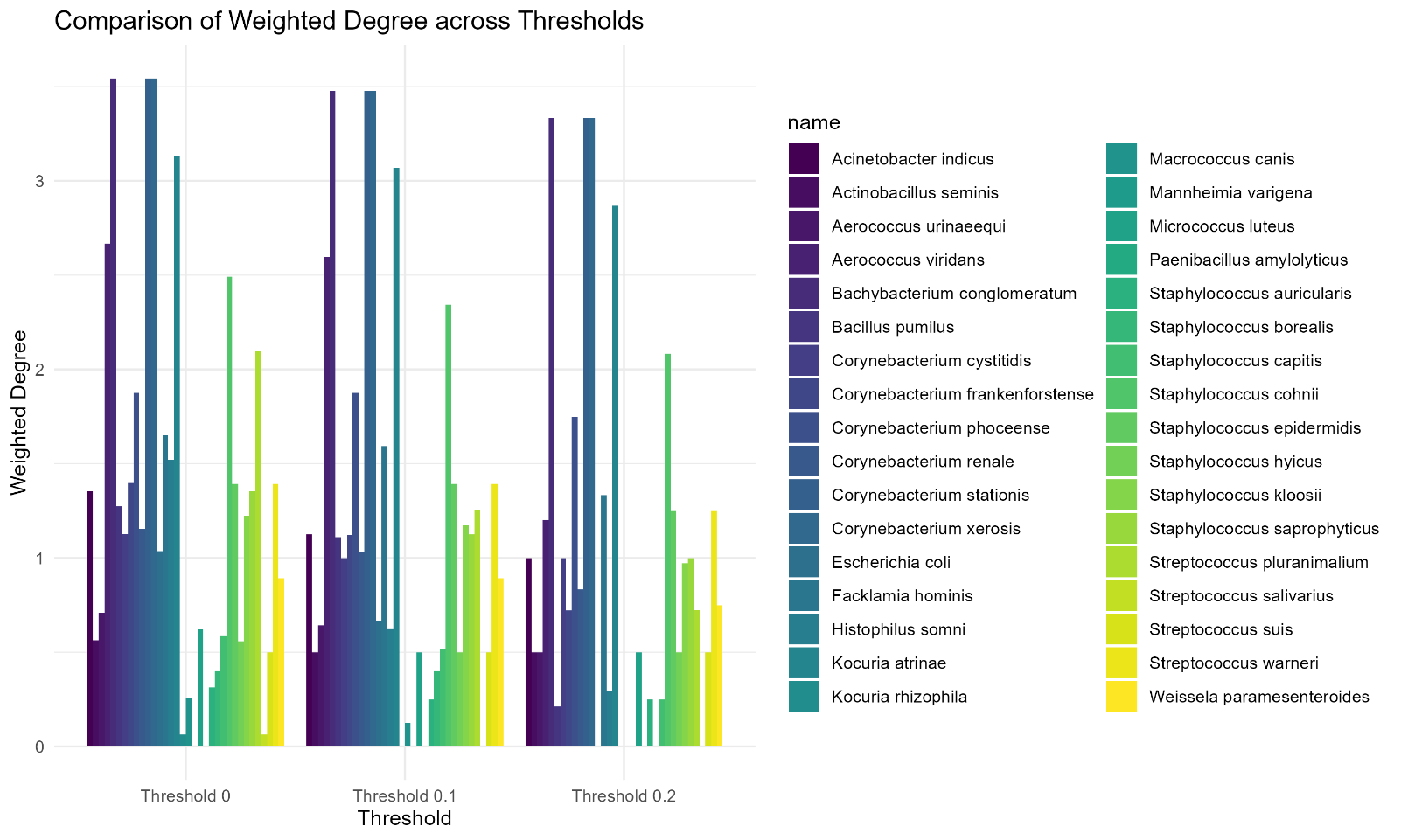

Supplement: Supplementary file 1 [file Table_1.DOCX]
